# Patterns of detectable viral load in a cohort of HIV‐positive adolescents on antiretroviral therapy in South Africa

**DOI:** 10.1002/jia2.25474

**Published:** 2020-03-17

**Authors:** Rebecca Sher, Sipho Dlamini, Rudzani Muloiwa

**Affiliations:** ^1^ Department of Paediatrics and Child Health University of Cape Town Cape Town South Africa; ^2^ Groote Schuur Hospital Cape Town South Africa; ^3^ Division of Infectious Diseases Department of Medicine University of Cape Town Cape Town South Africa

**Keywords:** adolescents, adherence, viral suppression, ARV, Sub‐Saharan Africa

## Abstract

**Introduction:**

Despite improved treatment and access to care, adolescent AIDS deaths are decreasing more slowly than in any other age group. There is lack of longitudinal data around adolescent adherence and the dynamics of viraemia over time. We aimed to describe patterns of detectable viral load (VL) in a cohort of adolescents attending an ARV clinic in Cape Town, South Africa.

**Methods:**

We conducted a retrospective cohort study of all patients on antiretroviral therapy aged 10 to 19 years.

Participants were included if they underwent at least two VL measurements and remained in care at the Groote Schuur Hospital HIV Clinic for at least 24 months between 2002 and 2016.

The primary outcome was two consecutive HIV VL >100 copies/mL, in line with the lower limit of detection of assays in use over the follow‐up period.

**Results and discussion:**

Of the 482 screened participants, 327 met inclusion criteria. Most participants had perinatally acquired HIV (n = 314; 96%), and 170 (52%) were males. Overall, there were 203 episodes of confirmed detectable VL involving 159 (49% (95% CI 43% to 54%)) participants during the follow‐up period. Six participants had genotyped resistance to protease inhibitors. Four of these never suppressed, while two suppressed on salvage regimens.

Total follow‐up time was 1723 person years (PY), of which 880 (51%) were contributed by the 159 participants who experienced detectable VL. Overall time with detectable VL was 370 PY. This comprised 22% of total follow‐up time, and 42% of the follow‐up time contributed by those who experienced detectable VL.

The rate of detectable VL was 11.8 (95% CI 10.3 to 13.5) episodes per 100 PY. The risk increased by 24% for each year of increasing age (Relative Risk 1.24 (95% CI 1.17 to 1.31); *p* < 0.0001).

There was no sex difference with respect to duration (*p* = 0.4), prevalence (*p* = 0.46) and rate (*p* = 0.608) of detectable VL.

**Conclusions:**

Clinicians need to be alert to the high prevalence of detectable VL during adolescence so as to pre‐empt it and act swiftly once it is diagnosed. This study helps to highlight the risk of detectable VL that is associated with increase in age as well the high proportion of time that poorly adherent adolescents spend in this state.

## 1 INTRODUCTION

AIDS‐related deaths in adolescents are decreasing much slower than in all other age groups despite improved treatment and access to care 1, 2. HIV/AIDS is one of the top ten leading causes of death in adolescents globally 1, 3, 4. This is thought to result from poor adherence to antiretroviral therapy (ART) 5, 6.

There is no gold standard for monitoring adherence to ART, but serial measurements of plasma HIV viral load (VL) are thought to give the best estimate 7, 8, 9. Resistance may also cause an increased VL, but in the context of effective ART, the HIV VL can be seen as both the outcome of poor adherence, and a sensitive means by which to monitor it 10, 11.

The consequences of ongoing detectable HIV viraemia include immune destruction, disease progression with higher risk of mortality, and greater risk of onward HIV transmission 5, 10, 12, 13, 14. There is evidence that even low‐level viraemia is associated with subsequent treatment failure and the development of resistance mutations 15, 16, 17, 18, 19.

Our understanding of health outcomes in adolescents with HIV is challenging due to a lack of disaggregated adolescent‐specific data 20. Our search found a paucity of longitudinal studies on adolescent adherence, with mostly cross‐sectional studies reporting only prevalence data. Our study describes the prevalence, incidence and duration of time spent with detectable VL in a cohort of HIV‐positive adolescents attending a public‐sector clinic in a low‐ and middle‐income (LMIC) setting.

## 2 METHODS

We conducted a retrospective cohort study using medical records of adolescents on ART in an established adolescent HIV clinical service in Cape Town, South Africa. The follow‐up period was from 1 January 2002 to 31 December 2016.

Groote Schuur Hospital's Adolescent HIV Clinic grew out of one of the first paediatric HIV clinics in South Africa. Most of the adolescents in care transitioned from the paediatric service at the same site, keeping the same clinical team comprised of doctors, nurses, lay‐counsellors, a social worker and a psychologist. It is located in a tertiary hospital and accepts referrals of adolescents from other paediatric services in the Cape Town metro. Transition to adult care takes place at age 20, provided the patient is no longer at school.

All adolescents aged 10 to 19 years of age who attended Groote Schuur Hospital's Adolescent HIV Clinic from 2002 (when the clinic began) to the end of 2016 were included if they had spent at least 24 months on ART after turning 10. Participants were followed until they were transferred out of the service, turned 20 years of age or until database closure.

The primary outcome was two consecutive detectable VLs of 100 copies/mL or more, taken at least two months apart. Over the entire period under review, clinical protocols dictated that VL be monitored annually in stable participants with undetectable VL, while VL measurements were repeated approximately two months after adherence intervention in participants with detectable VL >100 copies/mL. Where available, data on resistance genotyping were collated.

ART regimens including regimen switching were based on South African Department of Health guidelines. Participants on non‐nucleoside reverse transcriptase inhibitor (NNRTI)‐containing regimens were switched to a protease inhibitor (PI)‐containing regimen following two consecutive VL >1000 copies/ml taken at least two months apart. Genotyping was only performed on participants with VL consistently >1000 copies/mL while on a PI‐containing regimen for longer than two years. In the case of genotyped PI resistance, a darunavir/ritonavir containing “third‐line” regimen was selected by an expert committee. Participants who required rifampicin treatment for tuberculosis were given additional ritonavir boosting if on lopinavir/ ritonavir, while those on atazanavir/ritonavir used rifabutin‐based therapy 15.

Relevant information was extracted from medical records of all qualifying participants. The data extracted included demographic information, mode of HIV acquisition, date of diagnosis, date of first ART, ART regimens, VL measurements and mortality. Time lost to follow‐up was captured separately and contributed to the duration of time spent with detectable VL among participants who had confirmed detectable VL prior to interrupting care. Loss to follow‐up was defined as not returning to care for at least 12 weeks after the appointment date.

### 2.1 Data analysis

Data were directly entered electronically into a password protected database and then exported to STATA Version 14 (Stata Corporation, College Station, Texas, USA) to be checked and verified before analysis. Data were cleaned and queries were addressed using standardized approaches.

Categorical variables were depicted as percentages together with their 95% confidence intervals, as required. All continuous variables were summarized using medians with interquartile ranges (IQR).

The *χ^2^* test or Fisher's exact tests were used to assess the strength of association between two categorical variables as appropriate, while association between two continuous variables was tested with the Mann‐Whitney test.

The incidence of detectable VL was estimated by calculating the rate of primary outcome events over the time period the participants were in the study. Incidence was reported stratified by sex, age at outcome, age at ART initiation and length of time on ART. All rates were calculated per 100 person years (PY) and included 95% confidence interval estimates. To compare incidence between groups, unadjusted relative risk ratios and their 95% confidence intervals were estimated using the Mantel‐Haenszel method.

Participants who entered the study with detectable VL were categorized as having met the outcome at second consecutive detectable, VL while study outcomes following undetectable VL were regarded as separated events.

All participants meeting the inclusion criteria were enrolled. We estimated that at least 300 participants, with an average follow‐up period of five years, would be included.

A significance level was set at a two‐tailed *p* < 0.05 for all analysis.

A waiver of consent was granted by the Institutional Review Board. The protocol was approved by the Human Research Ethics Committee of the University of Cape Town (HREC REF: 899/2016).

## 3 RESULTS AND DISCUSSION

Of the 482 adolescents aged 10 to 19 years who attended clinic in the period under review, seven were not on ART for at least 24 months, while 148 attended the clinic for less than 24 months (this includes 91 participants who were transferred out or were lost to follow‐up before two years in care and 57 who had been adolescents for less than 24 months). This left 327 participants with sufficient data for analysis.

The cohort consisted of 170 (52%) males. The median age of joining the adolescent HIV clinic was 10.7 (IQR 10.0 to 12.8) years. Other baseline characteristics of the cohort are shown in Table 1. The number of patients followed up per calendar year increased over the follow‐up period, peaking in 2014 at 296 participants. HIV infection was acquired perinatally in 314 (96%) participants.

**Table 1:** Study baseline characteristics of HIV‐infected adolescents (N = 327)

Variable	n (%)/ Median (IQR)
Sex	
Male	170 (52)
Female	157 (48)
Mode of HIV acquisition	
Perinatal	314 (96)
Non‐perinatal	9 (2.8)
Unknown	4 (1.2)
Age (years)	
Study entry	10.6 (10.0 to 12.8)
Diagnosis	5.2 (1.3 to 9.1)
ART initiation	7.2 (3.2 to 9.9)
Study follow‐up time (years)	4.9 (3.6 to 6.6)
Duration of ART at entry (years)	4.4 (1.8 to 7.6)
ART naïve at entry	33 (10)
ART Regimen at entry	327 (100)
NNRTI	201 (61.5)
Protease Inhibitor	124 (37.9)
Holding	2 (0.6)
CD4 at entry (cells/μL)	710 (486 to 945)

ART, antiretroviral therapy; Holding, non‐ suppressive single drug regimen consisting of lamivudine; NNRTI, non‐nucleoside Reverse Transcriptase Inhibitor.

At baseline, 201 (62%) participants were on a regimen containing a NNRTI, 124 (38%) were on a PI and two (1%) were on a non‐suppressive “holding” regimen comprising lamivudine monotherapy with a background of known PI resistance. Prior virological failure resulting in a switch of drug class from NNRTI to PI had occurred in 73 (22%) of the participants.

### 3.1 Prevalence of detectable VL

A total of 2468 VL s were performed during the follow‐up period of which 885 (36%) were detectable at a threshold of >100 copies/mL. There were 159 (49% (95% CI 43% to 54%)) participants who experienced outcome‐defined confirmed detectable VL. At first detectable VL, the participants had a median age of 13.8 (IQR 11.2 to 15.5) years and had been on treatment for a median of 6.3 (3.4 to 9) years. A total of 86 (51%) of the 170 boys experienced confirmed detectable VL compared to 73 (47%) of the 157 girls; *p* = 0.46. See Table 2.

**Table 2:** Comparison by detectable viral load (VL) status

Variable	Confirmed detectable VL^a^ (n (%)/median (IQR)) n = 159	No confirmed detectable VL (n (%)/median (IQR)) n = 168	*p*
A. Study outcome confirmed detectable VL^a^ versus no confirmed detectable VL			
Male sex	86 (54)	84 (50)	0.460
Non‐perinatally acquired HIV	4 (3)	5 (3)	0.799
Age at ART initiation (years)	7.8 (4.3 to 10.2)	6.1 (2.6 to 9.6)	0.026
Age at study entry (years)	10.9 (10.0 to 13.6)	10.0 (10.0 to 12.5)	0.024
Follow‐up time in study (years)	5.1 (3.8 to 7.1)	4.7 (3.4 to 6.2)	0.021

Variable	At least one detectable VL (n (%)/median (IQR)) n = 216	Always undetectable VL (n (%)/median (IQR)) n = 111	*p*
B. At least one detectable VL (any VL> 100 copies/mL) versus always undetectable VL			
Male sex	114 (53)	56 (50)	0.690
Non‐perinatally acquired HIV	4 (2)	5 (5)	0.165
Age at ART initiation (years)	7.6 (3.4 to 10.1)	5.2 (2.8 to 9.4)	0.048
Age at study entry (years)	10.7 (10.0 to 13.0)	10.0 (10.0 to 12.6)	0.266
Follow‐up time in study (years)	5.1 (3.7 to 7.0)	4.7 (3.5 to 6.1)	0.063

Variable	Always detectable VL (n (%)/median (IQR)) n = 16	At least one undetectable VL (n (%)/median (IQR)) n = 311	*p*
C. Always detectable VL (>100 copies/mlL versus at least one undetectable VL			
Male sex	7 (44)	163 (52)	0.499
Non‐perinatally acquired HIV	1 (6)	8 (3)	0.381
Age at ART initiation (years)	7.9 (3.5 to 12.2)	7.1 (3.2 to 9.9)	0.395
Age at study entry (years)	13.2 (11.1 to 13.6)	10.3 (10.0 to 12.8)	0.007
Follow‐up time in study (years)	4.0 (3.6 to 5.5)	5.0 (3.6 to 6.7)	0.159

IQR, Interquartile Range; VL, viral load.

^a^ Confirmed detectable VL = At least two consecutive detectable VL >100 copies/mL.

Of the 159 participants who experienced detectable VL, 84 (53%) were on an NNRTI, 72 (45%) were on a PI and three (2%) were on a “holding regimen” at the start of detectable VL. Of these, 102 (64%) re‐suppressed. Of those that re‐suppressed, 61 (60%) did so without a change of drug class: 20 (20%) remained on an NNRTI, and 41 (40%) on a PI. The remaining 41 (40%) re‐suppressed following a change of drug class: 38 from NNRTI to PI, one from PI to NNRTI, and two from holding regimens to darunavir/ritonavir containing “third line regimens.”

Of the 102 who had re‐suppressed, 38 (37%) had a subsequent detectable VL. Six (16%) of those with subsequent detectable VL were on an NNRTI, and 32 (84%) were on a PI. Of these, 22 (58%) re‐suppressed, in 20 (91%) cases without a change of drug class (19 remained on a PI and one on an NNRTI). The remaining two (9%) re‐suppressed following a change of class from NNRTI to PI.

Of the 22 who had re‐suppressed for a second time, six (27%) went on to have a further detectable VL, all of whom were on a PI regimen.

Six of the 159 (4%) participants had genotyped resistance to PIs. Four of these never suppressed over the follow‐up time, while two entered the study with detectable VL, but later suppressed on darunavir/ ritonavir‐containing “third‐line” regimens.

Characteristics of the study cohort are compared by detectable VL status in Table 2.

### 3.2 Incidence of detectable VL

The 327 adolescents were followed up for a median of 4.9 (IQR 3.6 to 6.6) years. The total follow‐up time was 1723 PY of which 885 (51%) were contributed by males. The 159 participants who experienced detectable VL contributed 880 (51%) PY of the follow‐up time.

In total, 203 distinct episodes meeting the study outcome of two consecutive VL >100 copies/mL occurred with an overall rate of 11.8 (95% CI 10.2 to 13.5) per 100 PY over the follow‐up period.

**Figure 1:**
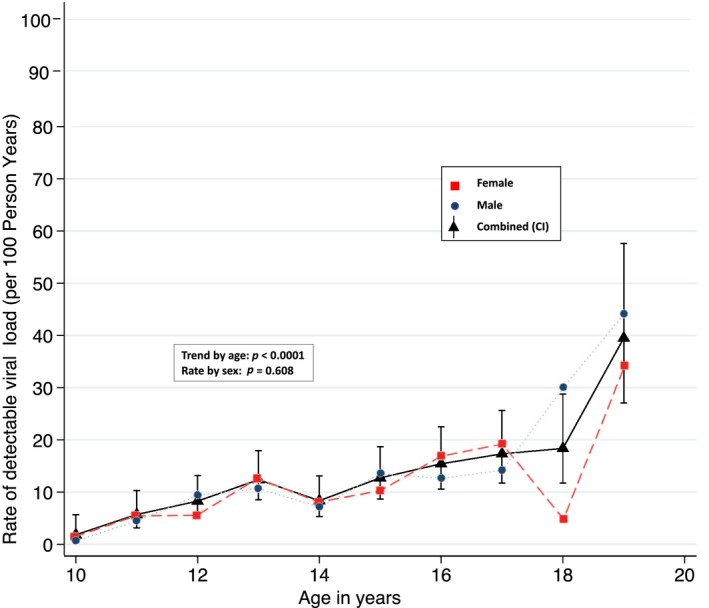
Rate of confirmed detectable viral load by age and sex.

The rate of detectable VL increased with increasing age with an overall relative risk of 1.24 (95% CI 1.17 to 1.31) for every additional year of adolescence (*p* < 0.0001). See Figure 1.

When the age of adolescents was categorized into age‐bands to fit with early, middle and late adolescence, participants aged 10 to 12 years had a rate of detectable VL of 5.6 (95% CI 3.9 to 7.9) per 100 PY while those aged 13 to 15 and 16 to 19 years had rates of 11.1 (95% CI 8.8 to 14) per 100 PY and 19.9 (95% CI 16.4 to 24.3) per 100 PY respectively.

The rate of detectable VL was 12.1 (95% CI 10.0 to 14.6) per 100 PY in males and 11.5 (95% CI 9.4 to 14.0) per 100 PY in females. The trend in the age rate was similar in males and females (*p* = 0.608). Figure 1.

The rate of detectable VL decreased with increasing time on ART. For participants who had been on ART for less than five years, the rate was 23.7 (95% CI 17 to 33) per 100 PY, while for those who had been on ART between five and ten years, the rate was 12.9 (95% CI 10.5 to 15.8) per 100 PY. The rate of detectable VL was lowest in participants who had been on ART for greater than 10 years, at 8.9 (95% CI 7.1 to 11.1) per 100 PY.

The risk of detectable VL decreased by 13% per each additional year on ART (unadjusted Relative Risk 0.87 (95% CI 0.83 to 0.91)).

### 3.3 Time spent with detectable VL

The 159 participants who experienced outcome‐defined detectable VL contributed 880 (51%) PY to the total follow‐up time of 1723 PY. Of the 880, 370 years were spent with detectable VL: this comprised 22% of the total follow‐up time, and 42% of the time contributed by participants who experienced detectable VL. Females contributed 173 (47%) of the 370 PY spent with detectable VL, while males contributed the remaining 197 (53%) years. Time with detectable VL comprised 21% of the total follow‐up time contributed by females, and 22% of that contributed by males; *p* = 0.4.

Participants experienced up to three separate episodes of detectable VL during the follow‐up period, separated by at least one VL <100 copies/ mL (Table 3). The median duration of the first episode was 1.5 (IQR 0.8 to 2.5) PY, of the second episode was 1.3 (IQR 0.8 to 2.5) PY and of the third episode was 1.1 (IQR 1.1 to 1.2) PY. In all cases, the third episode was ended by study exit.

**Table 3:** Proportion and duration of time spent with detectable viral load^a^

Duration (years)	n (%)
Total follow‐up time (Person Years)	1723 (100)
Males	885 (51)
Females	838 (49)
Follow‐up time (Person Years) contributed by participants with	
No detectable VL	843 (49)
Confirmed detectable VL	880 (51)
Time spent with detectable VL (Person Years)	370 (100)
Males	197 (53)
Females	173 (47)

Duration (years)	Median (IQR)
Total time (Person Years) with detectable VL, by number of episodes	
1 episode (n = 121)	1.5 (0.8 to 2.6)
2 episodes (n = 32)	2.8 (2.1 to 4.4)
3 episodes (n = 6)	3.5 (2.8 to 3.7)
Duration of suppression post detectable VL (Person Years)	
First suppression (n = 102)	1.3 (0.7 to 2.8)
Second suppression (n = 22)	0.9 (0.4 to 1.8)

VL, HIV viral Load.

^a^ Detectable viral load = Two consecutive viral loads >100 copies/mL.

Fifteen (4.6%) of the participants were lost to follow‐up and did not return to care within the study period.

Four (1.2%) of the 327 participants died during the study period: one died following trauma, while three succumbed to HIV‐related illness for a mortality rate of 0.2 per 100 PY (95% CI 0.08 to 0.6). All deaths occurred among participants with perinatally acquired HIV. The three HIV related deaths occurred in males. One death occurred at the age of 14 years and two at the age of 18 years. The youngest death occurred in a participant who did not meet criteria for confirmed detectable VL, as he had been virologically suppressed prior to a 19‐month period of loss to follow‐up immediately preceding his death. One of the participants who died at 18 years had detectable VL for the full 4.7 years of follow‐up. The other died with detectable VL after spending 6.2 of the 8.7 years of follow‐up in a state of detectable VL.

This study found that more than half of adolescents experience confirmed detectable VL. Although cumulative incidence of detectable VL averaged 12 per 100 PY over the adolescent period, it increased from 2 per 100 PY at the start of adolescence to 40 per 100 PY at 19 years of age. Most notably, although 22% of the whole cohort's follow‐up time was spent with detectable VL, those who ever experienced detectable VL spent 42% of their follow‐up time in this state.

Our results show a clear trend of increasing rates of detectable VL as the cohort ages. This is in keeping with findings from other studies that show older adolescents to be at higher risk of detectable VL 11, 21, 22, 23, 24, 25. Detectable VL was not sustained throughout the follow‐up period in a majority of adolescents, and showed a dynamic pattern of adherence and virological outcomes. The extremely high rate of detectable VL in the older adolescents within this cohort is of concern, as this occurs at the time prior to transition to adult care, which has been associated with poor health outcomes in both high and LMIC settings 26, 27, 28, 29.

The high prevalence of detectable VL found in this study is difficult to compare with other settings, as almost all identified studies are cross‐sectional, with widely varying results. Data from a private‐sector cohort in southern Africa found adolescents to be less adherent to ART, and to have lower rates of virological suppression and higher rates of virological rebound after initial suppression when compared to adults 30. A systematic review found adolescent virological suppression to range from 27% to 87% at any time since start of ART, while world‐wide adolescent adherence (based largely on VL, but also on self‐report) was estimated to be 62% 6, 31.

Our study contributes the seldom‐reported concept of “time spent with detectable VL” to the existing literature. We found that 22% of the total study time was spent with detectable VL >100 copies/mL. The average time spent with detectable VL in this study is lower than the 34% total time with detectable VL reported in the only other published study we could identify with a cohort of participants with perinatally acquired HIV 32. The study differed from ours in that the age of the cohort ranged from seven to thirty years of age and used a higher cut‐off of VL >400 copies/mL. Of concern is that when we restricted analysis of our data to the 159 participants who experienced detectable VL, the proportion of time spent with detectable VL rose to 42%. This indicates that those who have detectable VL tend to remain in this state for a considerable proportion of their follow‐up time. For adolescents, who may be sexually active, time spent with detectable VL should be viewed not only as time at greater risk of disease progression, but also time of increased risk of transmitting HIV 10, 13, 14.

Duration of detectable VL is rarely reported in the literature, possibly because of lack of longitudinal data. However, we believe it could be used as a potential marker of successful ART in adolescents. Although the duration of detectable VL can in part be affected by the frequency of virological testing, the frequent virological testing dictated by clinical protocols in those with detectable VL reduced the risk of overestimating this duration. We believe that when used together with incidence and prevalence data, duration of detectable VL has the potential to deepen our understanding of adherence patterns in adolescents on ART. Moreover it provides important additional information that is not reflected in prevalence and incidence estimates. This poorly‐explored concept may be useful in defining periods of particularly high risk of disease progression and transmission of HIV infection.

Although the quality of data was generally good, the retrospective design of the study meant that not all the data we would have liked was readily available, in particular that pertaining to HIV genotyping. While six participants were found to have resistance to PI‐containing regimens, it is possible that others also experienced undiagnosed resistance, and ineffective ART may have been responsible for persistent detectable VL in their cases.

## 4 CONCLUSIONS

Clinicians need to be alert to the high prevalence of detectable VL during adolescence so as to pre‐empt it and act swiftly once it has been diagnosed. This study helps to highlight the risk of poor adherence that is associated with increase in age as well the significantly high proportion of time that poorly adherent adolescents spend in this state.

## Competing interests

The authors declare that they have no competing interests.

## Authors' contributions

RS and RM conceptualized and designed the study. SD reviewed the study proposal. RS collected and cleaned data. RM oversaw statistical analyses and all authors interpreted results. RS led the drafting of the manuscript, while both RM and SD reviewed the manuscript. All authors have read and approved the final manuscript as submitted.
